# Risk factors for nonunion of osteoporotic vertebral compression fracture: a case‒control study

**DOI:** 10.1186/s12891-024-07386-1

**Published:** 2024-04-16

**Authors:** Shichuan Liao, Yan Xu, Jing Liu, Ling Jiang, Guogang Dai, Yi Wang

**Affiliations:** 1 Cervicodynia/Omalgia/Lumbago/Sciatica Department 2, Sichuan Province Orthopedic Hospital, No. 132 West First Section First Ring Road, Wuhou District, Chengdu, 610041 Sichuan Province China; 2 Experiment Teaching Center for Preclinical Medicine, Chengdu Medical College, No. 783, Xindu Avenue, Xindu District, Chengdu, Sichuan Province China; 3 College Hospital, Sichuan Agricultural University-Chengdu Campus, No. 211 Huiming Road, Wenjiang district, Chengdu, Sichuan Province China

**Keywords:** Osteoporotic vertebral compression fracture, Nonunion, Risk factors, Case‒control study

## Abstract

**Background:**

Early assessment of the risk of nonunion in osteoporotic vertebral compression fracture (OVCF) is beneficial to early clinical decision making. However, a comprehensive understanding of the risk factors for OVCF nonunion is lacking.

**Methods:**

We conducted a case–control study to investigate risk factors for OVCF nonunion. Patients who underwent surgery for nonunited OVCFs between January 2011 and December 2021 were eligible for inclusion as cases. Patients with successful OVCF healing confirmed by MRI over the same period were identified as controls. Patient demographics, comorbidities, and fasting blood test data were extracted for analysis.

**Results:**

A total of 201 patients with nonunited OVCFs and 1044 controls were included to evaluate the risk factors for nonunited OVCFs. There were statistically significant differences in sex, age, number of patients with hypertension, number of patients on bed rest after OVCF and T-score of BMD between the two groups. Logistic regression showed that female patients had a higher risk of OVCF nonunion than male patients and that smoking, drinking, diabetes, and hypertension were risk factors for nonunion of OVCFs, while bed rest and spinal support were protective factors against nonunion of OVCFs. We also found that age, BMD, FBG, and β-CTX were positively correlated with nonunited OVCFs, and that HGB and 1,25-(OH)2VitD3 level were negatively correlated with nonunited OVCFs.

**Conclusion:**

Smoking, drinking, diabetes and hypertension were risk factors for nonunion of OVCFs, while bed rest and spinal support were protective factors against nonunion of OVCFs. Age, BMD, FBG and β-CTX were positively correlated with nonunited OVCFs, while HGB and 1,25-(OH)2VitD3 level were negatively correlated with nonunited OVCFs. Based on the results of our study, we suggest that bed rest or spinal support for at least 3 consecutive weeks is necessary to reduce the risk of OVCFs nonunion.

## Background

Patients with osteoporosis are often prone to osteoporotic fractures due to decreased bone mass, destruction of the bone microstructure, increased bone fragility and decreased bone strength [1, 2]. Osteoporotic fractures, also called “fragility fractures”, occur in 50% of women and in 20% of men who are older than 50 years of age [3]. Osteoporotic vertebral compression fractures (OVCFs) are the most common type of osteoporotic fractures in middle-aged and elderly people [3]. OVCFs may cause pain and severe disability, thereby reducing the life expectancy of the patient [4, 5]. The mortality rate of long-term bedridden patients with OVCFs can reach 20%, and the permanent disability rate can reach 50% [3, 6, 7]. In 2010, there were 1.11 million people with OVCFs in China, costing 10 billion US dollars, and it is predicted that by 2050, there will be 3 million people with OVCFs in China, and the corresponding national medical cost will be 22 billion US dollars [8]. 37% of OVCFs are nonunited [9], thus leading to chronic low back pain, kyphosis, motor dysfunction, delayed nerve injury, etc., and greatly reducing the quality of life and even the life expectancy of patients.

Management of OVCFs includes surgical and nonsurgical treatments [10]. In terms of surgery, kyphoplasty and vertebroplasty are effective [11], enabling quick restoration of the weight-bearing function of the spine [12]. However, there is a risk of surgical complications [13]. Nonsurgical treatments usually require a bedridden period of 4–6 weeks, and nonunion is usually diagnosed months after primary OVCF. For patients with nonunited OVCFs, the nonsurgical treatment they receive is often ineffectual. Therefore, early assessment of the risk of nonunion is helpful for early surgical decision-making, avoiding ineffective nonsurgical treatment and improving clinical outcomes. However, a comprehensive understanding of the risk factors for OVCF nonunion is lacking, and no studies have investigated whether the commonly used clinical blood indices can be used to assess the risk of nonunion for OVCF. We included the commonly used clinical blood index for this case‒control study to investigate the risk of nonunion in OVCF.

## Methods

### Study design

We conducted a case‒control study to investigate the risk factors for OVCF nonunion using data from the Integrated Platform for Clinical Research (IPCR) of Sichuan Province Orthopedic Hospital, which includes more than 1.2 million records of demographic information, imaging data, laboratory data, diagnosis and treatment for patients treated at Sichuan Province Orthopaedic Hospital from 2011 to the present.

### Selection of cases and controls

Patients with a recorded diagnosis of nonunited OVCFs who underwent surgery between January 2011 and December 2021 were eligible to be selected as cases. Nonunited OVCFs were characterized by (1). Progressive back pain and kyphosis after an asymptomatic or mild symptomatic period regardless of history of spinal trauma; (2). Bone marrow edema in vertebral body on magnetic resonance imaging(MRI) regardless of history of any spinal trauma 6 months after the onset of back pain, or vertebral collapse, intravertebral vacuum cleft sign and intravertebral pseudoarthrosis presented on X-ray or computed tomography(CT) and “liquid sign” and “double contour sign” on MRI. Patients identified in the same period as the case group with successful OVCF healing confirmed by MRI were identified as controls. The control group was composed of (1). Patients with no vertebral collapse, intravertebral vacuum cleft sign or intravertebral pseudoarthrosis on X-ray or computed tomography scan; (2). Patients with no “liquid sign” or “double contour sign” on MRI. (3). Patients with no bone marrow edema in the vertebral body that could be seen on MRI. For both groups, we excluded patients with vertebral fractures caused by high-energy trauma, patients with coinfection, tuberculosis, or tumors at the time of presentation, patients with rheumatic immune diseases, patients with a history of spinal surgery, patients with a history of artificial prosthesis replacement or a history of blood transfusions, and patients younger than 40 years of age. This study was approved by the Ethics Committee of the Sichuan Province Orthopedic Hospital. All patients gave their informed consent prior to inclusion.

### Variables

We extracted patient demographic data, medical records, comorbidities, and fasting blood test data from the IPCR for both groups. The patient demographic data included age, sex, smoking status and drinking status. Medical record data included whether patients were on bed rest in a prone, lateral, or supine position for 24 h a day for more than 3 consecutive weeks after onset after OVCF, whether they underwent any form of rehabilitation, whether they used any spinal support devices for more than 3 consecutive weeks, whether patients had taken any form of calcium supplements (and the dosage, if applicable) for more than 3 months a year, and the average T-score for lumbar vertebrae extracted as the bone mineral density (BMD) index. Comorbidities included hypertension and diabetes. Fasting blood test data collected from both groups within one month of onset included routine blood markers, fasting blood glucose(FBG), serum electrolytes(Na, K, Ca, Mg, P), bone metabolism markers(bone gla protein(BGP), type I collagen carboxyl terminal peptide (beta-CTX) procollagen I N-Terminal propeptide(PINP)), 1,25-(OH)2VitD3, serum alkaline phosphatase (ALP), serum adenosine deaminase (ADA), serum γ-glutamyltransferase (γ-GGT), serum creatinine, serum total cholesterol (TC), triglycerides (TG), low-density lipoprotein cholesterol (LDL-C), high-density lipoprotein cholesterol (HDL-C), serum sodium (Na), serum phosphorus (P), serum magnesium(Mg), and C-reactive protein(CRP).

### Statistics

Statistical analysis was performed using SPSS22.0 software (Chicago, IL, USA). Continuous variables are presented as the means ± standard deviations (SDs). Multivariate imputation was used to impute missing data. Descriptive statistics were calculated for both groups. The t test was used to evaluate differences between continuous variables, and the χ2 test was used to evaluate differences between categorical variables. All tests were 2-sided, and *p* < 0.05 was defined as statistically significant. The risk factors for OVCF nonunion were evaluated by nonconditional logistic regression, and odds ratios (ORs) and 95% confidence intervals (CIs) were obtained.

## Results

**Fig. 1:**
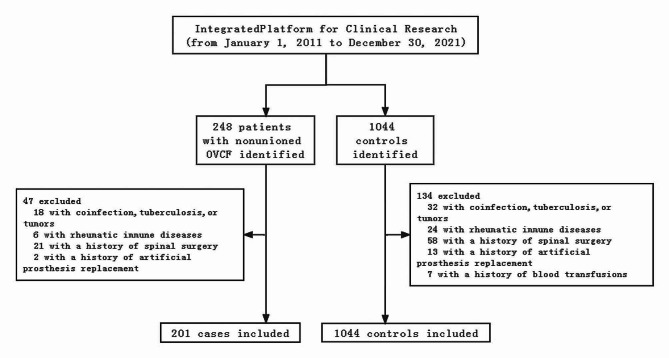
Flowchart of patient and matched control selection in the study

A total of 248 patients who were diagnosed with OVCF nonunion and who underwent surgery and 1178 patients with successful OVCF healing confirmed by MRI were identified between January 2011 and December 2021. Forty-seven patients with nonunited OVCFs and 134 patients with successful OVCF healing were excluded, and 201 patients with nonunited OVCFs and 1044 controls were included to evaluate the risk factors for OVCF nonunion (Fig. 1).

Demographics and comorbidities of the participants are shown in Table 1. There were statistically significant differences in sex, age, number of patients with hypertension, number of patients who were on bed rest after OVCF and T-score of BMD between the two groups. There was no statistically significant difference in the number of smokers or alcohol drinkers, number of patients with diabetes, number of patients who had taken rehabilitation, number of patients who had used spinal support devices or the number of patients who took calcium between the two groups.

**Table 1:** Demographics and comorbidities of the participants

		Case(*N* = 201)	Control(*N* = 1044)	Statistics	*P*
Gender				χ²=4.67	0.03
	Male	26	318		
	Female	175	726		
Age		70.13 ± 8.88	66.47 ± 12.74	T=-3.90	0.00
Smoking				χ²=0.06	0.81
	Yes	30	163		
	No	171	881		
Drinking				χ²=1.12	0.29
	Yes	13	92		
	No	188	952		
Diabetes				χ²=3.12	0.08
	Yes	28	102		
	No	173	942		
Hypertension				χ²=8.23	0.00
	Yes	69	257		
	No	132	787		
Bed rest				χ²=14.39	0.00
	Yes	98	658		
	No	103	386		
Rehabilitation				χ²=0.81	0.37
	Yes	129	704		
	No	72	340		
Spine support				χ²=1.00	0.32
	Yes	146	793		
	No	55	251		
Calcium intake				χ²=0.76	0.39
	Yes				
	No				
T-score		-3. 82 ± 1. 29	-3. 27 ± 0. 84	T = 2.36	0.02

Laboratory data of the participants are shown in Table 2. There were statistically significant differences in HGB, Mg, β-CTX, 1,25-(OH)2VitD3 and ADA between the two groups, while there was no statistically significant difference in the white blood cell count, red blood cell count, FGB, Na, K, Ca, P, BGP, PINT, ALP, γ-GGT, creatinine clearance(Ccr), TC, TG, LDL-C, HDL-C, or CRP between the two groups (Table 2).

**Table 2:** Clinical blood indices of the participants

Variable	Case(*N* = 201)	Control(*N* = 1044)	T	*P*
WBC	6.21 ± 2.47	6.45 ± 2.11	1.45	0.15
RBC	4.06 ± 0.57	4.14 ± 0.58	1.75	0.08
HGB	121.74 ± 15.98	125.29 ± 16.95	2.74	0.00
FBG	5.39 ± 1.12	5.39 ± 1.12	0.27	0.76
Na	141.24 ± 2.94	141.25 ± 2.85	0.04	0.97
K	4.04 ± 0.76	3.98 ± 0.77	1.42	0.17
Ca	2.20 ± 0.05	2.21 ± 0.05	-0.74	0.47
Mg	0.87 ± 0.09	0.89 ± 0.84	3.41	0.00
P	1.16 ± 0.20	1.16 ± 0.19	-0.13	0.90
BGP	7.72 ± 3.74	7.3 + 4.01	-1.38	0.42
β-CTX	0.80 ± 0.12	0.52 ± 0.07	9.22	0.00
PINP	70.07 ± 5.26	71.46 ± 6.05	-0.94	0.33
1,25-(OH)2VitD3	8.82 ± 1.10	15.59 ± 2.24	5.66	0.00
ALP	89.25 ± 46.23	89.03 ± 40.43	-0.07	0.95
ADA	10.63 ± 4.17	9.93 ± 4.19	-2.16	0.03
γ-GGT	33.10 ± 45.79	32.94 ± 47.95	-0.04	0.97
Ccr	63.12 ± 20.29	61.90 ± 17.93	0.55	0.61
TC	4.59 ± 1.12	4.64 ± 0.98	0.58	0.56
TG	1.51 ± 0.86	1.42 ± 0.89	-1.26	0.21
LDL-C	2.44 ± 0.79	2.46 ± 0.76	0.21	0.81
HDL-C	1.40 ± 0.37	1.39 ± 0.34	-0.12	0.90
CRP	11.37 ± 27.86	11.57 ± 24.06	0.10	0.92

Parameters with statistically significant differences between the two groups were included in the logistic regression analysis, as well as factors that were thought to have an effect on fracture healing, such smoking, drinking diabetes and FBG. Although rehabilitation and spinal support did not show statistically significant differences between the two groups, these two factors were included in the logistic regression because they are common clinical interventions. Rehabilitation for all included cases in this study included medium-frequency pulse treatment, wax therapy, polarized light therapy, ultrasonic therapy, and spinal extension exercise. The nonconditional logistic regression showed that female sex (OR, 1.73; 95% CI, 1.26–1.97),, smoking (OR, 1.96; 95% CI, 1.22–3.52), drinking (OR, 1.74; 95% CI, 1.02–2.85), diabetes (OR, 2.03; 95% CI, 1.04–3.67), and hypertension (OR, 1.32; 95% CI, 1.06–1.84) were risk factors for nonunion of OVCFs, and that bed rest (OR, 0.53; 95% CI, 0.21–0.93) and spinal support (OR, 0.82; 95% CI, 0.73–0.96) were protective factor against nonunion of OVCFs. We also found that age (OR, 1.25; 95% CI, 1.03–1.82), BMD (OR, 2.23; 95% CI, 1.97–3.12), FBG (OR,1.07; 95% CI, 1.00-1.87), β-CTX (OR, 1.01; 95% CI, 0.99–1.08) were positively correlated with nonunited OVCFs, and that HGB (OR, 0.94; 95% CI, 0.90–0.99) and 1,25-(OH)2VitD3 level (OR, 0.93; 95% CI, 0.89–0.99) were negatively correlated with nonunited OVCFs(Table 3).

**Table 3:** Risk factors for osteoporotic vertebral compression fracture nonunion

Variate	SE	*P*	OR	95% CI
Gender	0.19	0.02	1.73	1.26–1.95
Age	0.19	0.00	1.25	1.03–1.82
smoking	0.13	0.02	1.96	1.22–3.53
Drinking	0.12	0.02	1.74	1.02–2.85
Diabetes	0.71	0.00	2.03	1.04–3.67
Hypertension	0.18	0.04	1.32	1.06–1.84
Bed rest	0.13	0.02	0.53	0.21–0.93
Rehabilitation	0.24	0.12	0.87	0.81–0.97
Spine support	0.11	0.02	0.82	0.73–0.96
T-score	0.75	0.00	2.23	1.97–3.12
HGB	0.16	0.01	0.94	0.90–0.99
FBG	0.68	0.01	1.07	1.00-1.87
β-CTX	0.01	0.03	1.01	0.99–1.08
1,25-(OH)2VitD3	0.16	0.00	0.93	0.89–0.99
Mg	0.13	0.16	0.67	0.12–0.82
ADA	0.18	0.20	1.05	0.99–1.07

Logistic regression showed that bed rest (OR, 0.53; 95% CI, 0.21–0.93) and spinal support (OR, 0.82; 95% CI, 0.73–0.96) were protective factors against OVCF nonunion. Bed rest and spinal support are practical for patients and are usually used as early pain relief interventions; thus, we further analyzed these two factors in a subgroup analysis (Table 4).

**Table 4:** Subgroup analysis of risk factors for nonunion of OVCFs

Subgroup	Case(%)	Control(%)	χ²	*P*
	*N* = 201	*N* = 1044		
**Bed rest**				
**No**	73(36.32)	202(19.35)	125.82	0.00
< 3weeks	44(21.89)	231(22.13)	2.796	0.09
3-6weeks	53(26.37)	379(36.30)	46.78	0.00
> 6weeks	31(15.42)	232(22.22)	19.97	0.00
**Spine support**				
**No**	83(41.29)	211(20.21)	113.74	0.00
< 3weeks	39(19.40)	286(27.39)	39.45	0.00
3-6weeks	49(24.38)	343(32.85)	40.27	0.00
> 6weeks	30(14.93)	204(19.54)	4.689	0.03

## Discussion

OVCF is a common fracture in middle-aged and elderly people, and nonunion is a common complication of OVCF. Avascular necrosis is the main mechanism of OVCF nonunion. Nonunited OVCFs may prolong the duration of pain, aggravate the degree of pain, increase the need for the patient to remain bedridden, increase the occurrence of cognitive impairment, cause delayed intravertebral cleft with neurological symptoms, and greatly reduce the patient’s quality of life [14–16]. Early assessment of the risk of OVCF nonunion is helpful for reducing the incidence of ineffective nonsurgical treatments and for facilitating early decision-making. However, there has been little research on the risk factors for OVCF nonunion at present.

Shunsuke [17] reported that the formation of endplate cysts is a predictive factor of OVCF nonunion. However, despite the above studies, research on risk factors for OVCF nonunion remain nonextensive and insufficient.

This case‒control study was conducted to investigate the risk factors for OVCF nonunion. OVCF is secondary to osteoporosis. Patients who are younger than 40 years of age rarely develop osteoporosis, which is usually secondary to other diseases or medication use. Patients who are younger than 40 years of age usually sustain a vertebral compression fracture as a result of high-energy trauma, and in some accidental cases, vertebral compression fractures may present in people under 40 with normal bone mineral density who experience minor trauma. So, we excluded patients under 40 to avoid bias in the study results. The risk factors for nonunion of OVCFs included smoking, drinking, diabetes and hypertension. Smoell et al. reported that smoking increased the risk of fracture nonunion with an overall risk ratio(RR) of 1.89 (95% CI: 1.60–2.24) [18]. As a risk factor for fracture nonunion, smoking has been widely reported with a RR of 1.13(95% CI: 1.60–2.24) for tibial fracture [19], with a RR of 3.64 (95% CI: 1.23–-10.07) for intertrochanteric femoral fracture [20], with a RR of 2.23 (95% CI: 0.71–6.97) for forearm fracture [21], with a RR of 1.51 (95% CI: 0.73–3.13) for distal femoral fracture [22], with a RR of 1.72 (95% CI: 1.15–2.58) for thoracolumbar fractures [23]. Akanksha et al. found that smoking increased the risk of nonunion of tibial shaft fractures(OR, 1.45; 95% CI, 1.06–1.98), as well as delayed union(OR, 2.19; 95% CI, 1.22–3.93) [24]. Mundi found that the rate of tibial shaft fracture nonunion in active smokers was higher than that in non-smokers [25]. Although drinking alcohol may affect fracture healing, there is not much literature to support this view. A meta-analysis failed to include any study comparing the rates of delayed union between alcohol consumers and non- alcohol consumers; however, it was found that there was no significant difference in the non-union rates between the two groups [26]. In another study, a history of alcohol was reported to be a risk factor for non-union of fractures of the ankle and metatarsals, with an OR of 2.7 (95% CI, 1.06–1.98) [27]. Askew et al. reported that the time to union in patients who drink alcohol was longer than that in non-drinkers [28]. Zura et al. reported that alcohol consumption was a risk factor for non-union, with an overall OR of 1.05(95% CI, 0.94–1.17), and that diabetes was a risk factor for non-union of trunk fractures, with an OR of 1.67 (95% CI, 1.15–2.41) [29]. Lavery et al. found that the rate of nonunion of ankle fractures in patients with diabetes was up to 24.5% [30]. The relationship between hypertension and fracture nonunion has not been reported. We also found that there are no reports on bed rest being a protecting factor for nonunion of OVCFs.

Our study revealed that age, BMD, FBG, β-CTX were positively correlated with nonunited OVCFs. It has been noted that the risk of trunk fracture nonunion increases with age, with an OR of 1.03 (95% CI, 0.93–1.13) [29]. One study reported that BMD was independently associated with delayed union of vertebral fractures following percutaneous kyphoplasty, but the role of BMD in the nonunion of non-surgically treated OVCFs has not been reported [31]. Although diabetes has been proven to be positively correlated with nonunited fractures [29, 30], the effect of an elevated FBG on OVCF nonunion remains unknown. β-CTX was positively correlated with OVCF nonunion [32, 33], but the relationship betweenβ-CTX and nonunited OVCFs has not been investigated. Our results also revealed that HGB and 1,25-(OH)2VitD3 level were negatively correlated with nonunited OVCFs(Table 3). A multicenter evaluation revealed that decreased HGB was associated with fracture nonunion [34]. Decreased HGB was also found to be associated with fracture nonunion after surgery [35]. Vitamin D deficiency was reported to be a risk factor for nonunited OVCFs, with an OR of 1.14 (95% CI, 1.05–1.22) [32]. These reports were consistent with our findings.

The present study showed that OCVF nonunion was more likely in women than men, with an OR of 1.73 (95% CI, 1.26–1.97), andwe investigated common basic diseases and commonly used clinical blood indices as risk factors for OVCF nonunion. We found that sex, smoking, drinking and diabetes were not significantly correlated with OVCF nonunion. We identified age and hypertension as risk factors for OVCF nonunion, with ORs of 1.02 (95% CI, 1.00-1.03) and 1.41 (95% CI, 1.01–1.99), respectively. We also found that the serum levels of Mg and HGB were negatively correlated with OVCF nonunion, with ORs of 0.07 (95% CI, 0.01–0.41) and 0.99 (95% CI, 0.98-1.00), respectively. Bed rest (OR, 0.53; 95% CI, 0.21–0.93) and usage of spinal support (OR, 0.82; 95% CI, 0.73–0.96) helped reduce the risk of OVCF nonunion.

Our study had limitations. The present work was a database-based retrospective study, the data were extracted from the IPCR of Sichuan Province Orthopedic Hospital, and was limited by the structure of the database. There are still risk factors that are potentially related to the nonunion of OVCFs which were not investigated in our work, including essential characteristics, comorbidities, medication history, nutrition status, etc. More in-depth and comprehensive studies on the importance of an early assessment of the risk of nonunion of OVCFs are needed in the future. Based on the results of logistic regression and subgroup analyses, we suggest that bed rest or spinal support for at least 3 consecutive weeks of is necessary to reduce the risk of OVCF nonunion.

## Conclusions

In conclusion, our study showed women had a higher risk of OVCF nonunion than men and that smoking, drinking, diabetes and hypertension were risk factors for nonunion of OVCFs, and that bed rest and spinal support were protective factors against nonunion of OVCFs. We also found that age, BMD, FBG and β-CTX were positively correlated with nonunited OVCFs, and that HGB and 1,25-(OH)2VitD3 level were negatively correlated with nonunited OVCFs. We suggest that bed rest or spinal support for at least 3 consecutive weeks is necessary to reduce the risk of OVCFs nonunion. We hope our findings will be helpful for assessing the risk of OVCF nonunion, and we hope our study will provide a reference for future studies on the risk factors for nonunion of OVCFs.

## Data Availability

Data from the Integrated Platform for Clinical Research (IPCR) of Sichuan Province Orthopedic Hospital is available from the corresponding author.

## Abbreviations

β-CTX: Type I collagen carboxyl terminal peptide
γ-GGT: γ-glutamyltransferase
ADA: Adenosine deaminase
ALP: Alkaline phosphatase
BMD: Bone mineral density
BPG: Bone gla protein
CIs: Confidence intervals
CRP: C-reactive protein
CT: Computed tomography
FBG: Fasting blood glucose
HDL-C: High-density lipoprotein cholesterol
HGB: Hemoglobin
IPCR: Integrated Platform for Clinical Research
LDL-C: Low-density lipoprotein cholesterol
Mg: Serum magnesium
MRI: Magnetic resonance imaging
Na: Serum sodium
ORs: Odds ratios
OVCF: Osteoporotic vertebral compression fracture
P: Serum phosphorus
PINP: Procollagen I N-Terminal propeptide
TC: Serum total cholesterol
TG: Triglycerides
